# Evaluation of Spinal Cord Blood Supply with Hyperspectral Imaging of the Paraspinous Musculature During Staged Endovascular Repair of Thoracoabdominal Aortic Aneurysm: A Sub-Study of the Prospective Multicenter PAPA-ARTiS Trial

**DOI:** 10.3390/jcm14093188

**Published:** 2025-05-05

**Authors:** Birte Winther, Daniela Branzan, Christian D. Etz, Antonia Alina Geisler, Sabine Steiner, Hinrich Winther, Raphael Meixner, Marina Jiménez-Muñoz, Hannes Köhler, Dierk Scheinert, Andrej Schmidt

**Affiliations:** 1 Clinic of Angiology, University Hospital Leipzig, 04103 Leipzig, Germany; dierk.scheinert@medizin.uni-leipzig.de (D.S.); andrej.schmidt@medizin.uni-leipzig.de (A.S.); 2 Helmholtz Institute for Metabolic, Obesity and Vascular Research (HI-MAG), The Helmholtz Zentrum Munich, The University of Leipzig and University Hospital Leipzig, 04103 Leipzig, Germany; daniela.branzan@mri.tum.de (D.B.); sabine.m.steiner@meduniwien.ac.at (S.S.); 3 Department of Vascular and Endovascular Surgery, Rechts der Isar Hospital, 81675 Munich, Germany; 4 Department of Cardiac Surgery, University Medicine Rostock, 18057 Rostock, Germany; christian.etz@med.uni-rostock.de; 5 Clinical Department of General, Visceral and Transplant Surgery, University Hospital Graz, 8036 Graz, Austria; antonia.geisler@medunigraz.at; 6 Division of Angiology, Department of Internal Medicine II, Medical University Vienna, 1090 Vienna, Austria; 7 Institute of Diagnostic and Interventional Radiology, Medizinische Hochschule Hannover, 30625 Hannover, Germany; winther.hinrich@mh-hannover.de; 8 Core Facility Statistical Consulting Helmholtz Munich, 85764 Neuherberg, Germanymarina.jimenezmunoz@helmholtz-munich.de (M.J.-M.); 9 Innovation Center Computer Assisted Surgery (ICCAS), Faculty of Medicine, University of Leipzig, 04103 Leipzig, Germany; hannes.koehler@medizin.uni-leipzig.de

**Keywords:** collateral network, aortic aneurysm, spinal cord ischemia, endovascular repair, hyperspectral imaging

## Abstract

**Background/Objectives**: Our aim was to assess the feasibility of hyperspectral imaging (HSI) to detect changes in tissue oxygenation (StO_2_) of the back, as non-invasive spinal cord collateral network (CN) monitoring during staged endovascular repair (ER) of thoracoabdominal aortic aneurysm (TAAA). **Methods**: Between September 2019 and June 2021, 20 patients were treated for TAAA and underwent HSI. They were randomized 1:1 to minimally invasive staged segmental artery coil embolization (MIS2ACE) (*n* = 10) and staged stentgraft implantation (*n* = 10) as priming methods. HSI of paravertebral regions was taken during each procedure and up to 10 days after. The primary endpoint was the identification of StO_2_ changes after ER of TAAA. **Results**: TAAA Crawford Type II (*n* = 17) and Type III (*n* = 3) were treated. After stentgrafting, StO_2_ increased immediately (*p* < 0.001), followed by a decrease after 5 days (*p* < 0.001) and 10 days (*p* = 0.028). StO_2_ was significantly higher in the thoracic compared to the lumbar region. There was no significant difference between MIS2ACE and the first stentgrafting for StO_2_ (*p* = 0.491). Following MIS2ACE, definitive ER caused a significant decrease in StO_2_ after 5 days (*p* = 0.021), which recovered to baseline after 10 days (*p* = 0.130). After stentgraft priming, definitive ER caused a significant decrease in StO_2_ after 24 h (*p* = 0.008), which did not return to baseline after 5 (*p* < 0.001) and 10 days (*p* = 0.019). **Conclusions**: HSI detected significant changes in StO_2_ in the thoracic and lumbar paravertebral regions during ER of TAAA. These preliminary data suggest the efficacy of MIS2ACE in priming the CN before ER of TAAA.

## 1. Introduction

Thoracoabdominal aortic aneurysm (TAAA) is characterized by the irreversible dilation of the descending thoracic and abdominal aorta, resulting from the degeneration of elastic fibers and vascular smooth muscle cells within the aortic wall [1]. Due to their clinically silent nature, determining the exact prevalence of TAAA is challenging, and patients often remain undiagnosed until rupture [1]. However, the risk of rupture increases with the size of the aneurysm, and rupture is associated with high mortality and morbidity, emphasizing the critical need for early diagnosis and aortic repair (AR) [2].

Endovascular repair (ER) of TAAA has shown reduced perioperative mortality and shorter recovery times compared to open repair (OR), making it the preferred treatment option for high-risk patients [3]. However, spinal cord ischemia (SCI), a severe complication of AR, remains a significant concern. Inadequate oxygen supply due to the abrupt occlusion of multiple segmental arteries (SAs) following stentgraft implantation causes SCI, which can lead to paraplegia and paraparesis, and is associated with increased morbidity and early mortality [4,5]. When feasible, the implantation of custom-made devices with shorter landing zones is preferred to minimize the risk of SCI [6]. Nevertheless, over 15% of TAAA patients undergoing ER still experience SCI, and protective strategies have been developed to further mitigate this risk [5].

Cumulative studies and clinical observations suggest that a robust collateral network (CN) must exist to explain the maintenance of spinal cord perfusion when SAs are interrupted during TAAA treatment [7]. The CN, primarily located in the paraspinous musculature, is fed by SAs and branches of the subclavian and hypogastric arteries [8]. This concept led to the introduction of staged AR, where SAs are closed in multiple steps, allowing the CN to remodel and sustain adequate spinal cord perfusion [9,10]. This can be achieved through staged stentgraft implantation or ‘minimally invasive staged segmental artery coil embolization’ (MIS2ACE), with both approaches demonstrating promising results in reducing SCI rates [9,10].

Nonetheless, monitoring of the CN remains crucial for spinal cord protection management. While motor-evoked potentials are reliable for assessing spinal cord functional integrity in perioperative settings, they are not feasible when the level of patients’ sedation is reduced postoperatively [11,12]. Late-onset SCI occurs in 60% of patients suffering from SCI after TAAA AR, highlighting the need for CN monitoring outside the operating room [5]. Lumbar near-infrared spectroscopy (NIRS) has been shown to effectively monitor tissue oxygenation (StO_2_) in the paraspinous musculature, serving as a surrogate for spinal cord oxygenation [13]. This non-invasive method demonstrated real-time responsiveness to SA occlusion and correlated with permanent neurologic deficits in animal models [14]. However, NIRS measurements have limitations due to the restricted number of optodes and their placement, making it challenging to assess paraspinous oxygenation during TAAA repair [15].

Hyperspectral imaging (HSI), a new non-invasive imaging method based on the principles of optical remission spectroscopy, offers an adjustable image area for measurement and may overcome these limitations [16]. The HSI camera emits light in the visible and near-infrared spectrum, which is partially absorbed by chromophores (e.g., oxygenated/deoxygenated hemoglobin) and scattered, with scattering assumed constant. A sensor then measures the wavelengths of the remitted light and uses these data to calculate various parameters, such as tissue oxygenation [16].

This proof-of-concept study aimed to evaluate the feasibility of HSI in monitoring the CN of the spinal cord during and up to ten days following ER of TAAA, using the paraspinous musculature as a surrogate. Additionally, the study sought to gain new insights into CN remodeling after priming with MIS2ACE and staged stent-graft implantation.

## 2. Materials and Methods

**Figure 1:**
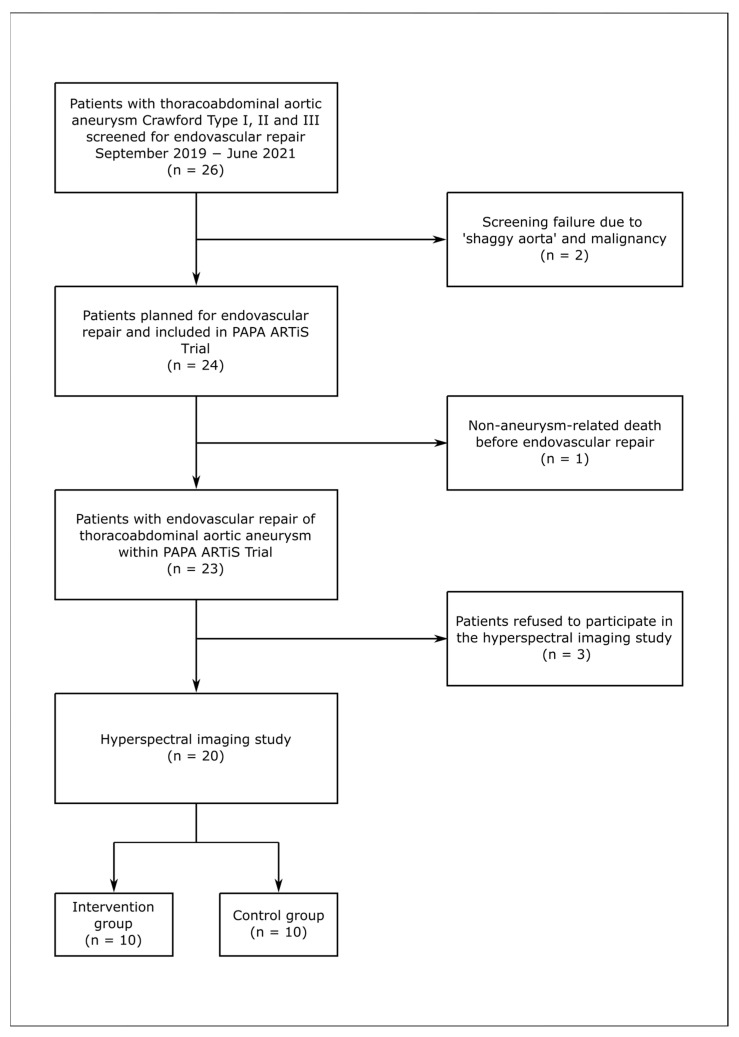
Study Flow Chart.

This prospective study enrolled patients diagnosed with TAAA who were scheduled for ER within the Paraplegia prevention in aortic aneurysm repair by thoracoabdominal staging with MIS2ACE Trial: PAPA ARTiS Trial (Figure 1). The PAPA ARTiS Trial was registered with clincaltrials.gov (NCT03434314), and a detailed trial protocol was published in 2019 [17]. The primary objective of this randomized trial was to evaluate whether MIS2ACE could reduce the incidence of SCI and mortality compared with standard TAAA repair alone. Patients were randomized to the interventional or the control arm. In the interventional arm, SAs were occluded prior to aneurysm repair, using the technique described previously [10]. In the control arm, treatment followed the standard procedure at the study site. Following approval of the PAPA ARTiS Trial (AZ 435/17), a sub-study was initiated to evaluate spinal cord perfusion using HSI after staged ER of TAAA with and without MIS2ACE. This sub-study was approved by the Institutional Review Board of the University of Leipzig (177/19-ek—8 July 2019), and patients gave written informed consent.

### 2.1. Inclusion and Exclusion Criteria

The inclusion and exclusion criteria were identical to the PAPA ARTiS Trial [17]. Inclusion criteria consisted of patients aged 18 years or older with TAAA of Crawford types I, II or III, scheduled for ER within four months. Exclusion criteria included complicated (sub)acute type B aortic dissection, ruptured or urgent aneurysms, untreated aortic arch aneurysms, bilateral occlusion of the iliac arteries or chronic total occlusion of the left subclavian artery, preoperative neurological deficits, major untreated cardiopulmonary disease, a life expectancy of less than one year, high risk for SA embolism (‘shaggy’ aorta), and severe contrast agent allergy or significant reduction in glomerular filtration rate.

### 2.2. Methodology of Conducted Research Procedures

**Figure 2:**
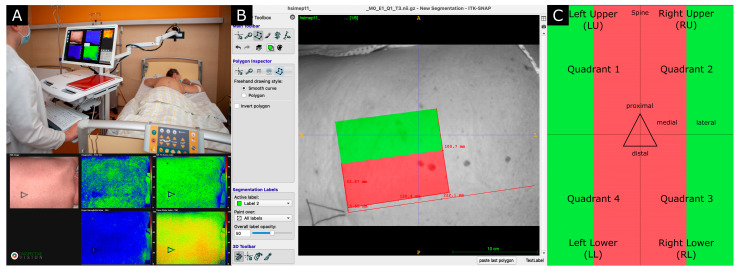
Hyperspectral imaging of the back of the patient. (**A**) Image acquisition with TIVITA^®^ Tissue camera system; (**B**) image post-processing with ITK-Snap; (**C**) delimitation of the regions of interest. The paravertebral region that overlaps with the paravertebral musculature is shown in red, whereas the non-overlapping region is shown in green.

Following randomization, HSI was performed in the prone position at five different time points: before the intervention (T1); directly after the intervention (T2); 24 h after the intervention (T3); 5 ± 1 days after the intervention (T4); 10 ± 2 days after the intervention (T5) (Figure 2). Perioperative management of these patients was presented elsewhere [10]. The primary endpoint was the identification of changes in StO_2_, measured with HSI in thoracic and lumbar paravertebral regions as indicators of ischemia perioperatively. HSI was performed with the TIVITA^®^ Tissue camera system (Diaspective Vision GmbH, Am Salzhaff-Pepelow, Germany). The camera measures the light reflected by tissue exposed to both visual and near-infrared light, calculating index parameters from the light spectra that are encoded into color maps for visualization [18]. The analysis software generates a red–green–blue color image and a false color image for each of the four parameters: StO_2_, tissue hemoglobin index (THI), near-infrared perfusion index (NIR PI) and tissue water index (TWI) (Figure 2A). The means and standard deviations for each examination were extracted using Python (version 3.7.8), a general-purpose programming language (Figure 2B). The patient’s back was divided into four quadrants, with only the paravertebral thoracic and lumbar regions evaluated due to their overlap on the paravertebral musculature (Figure 2C).

### 2.3. Statistical Analysis

As this was a pilot study, no formal sample size calculation was performed. Twenty patients were intended to be included. Demographics, medical history and procedure-related data were collected in an electronic database (REDCap 12.0.3).

Statistical analyses were performed using R-4.3.22. [19]. To capture the individual-specific patient effects and to account for the repeated measurements, a linear mixed model (lmm) with patient ID as a random intercept was performed using the lmer function from the lme4 package in R (version 1.1-35.1) with default options (REML = TRUE) [20]. MIS2ACE in the intervention arm and the first stentgraft implantation in the control arm were defined as priming methods. Staged ER was defined as the intentional completion of ER across separate sessions.

To test the feasibility of HSI in detecting StO_2_ changes (range, 0–1) during ER of TAAA, measurements acquired after stentgraft implantation without prior MIS2ACE were included (Model 1). To examine the effect of priming methods, all HSI measurements performed during MIS2ACE (intervention group), and the implantation of the first stentgraft (control group) were included (Model 2). To assess StO_2_ changes during ER of TAAA after priming the CN with MIS2ACE, HSI measurements of all the sessions of implantation of stentgrafts in the MIS2ACE group were used (Model 3). To measure the changes in StO_2_ after priming the CN with stentgrafts in the control group, HSI measurements from the following sessions were used (Model 4). In all models, HSI measurements were the response variable, and time and region were used as categorical variables. Time point T1, Region R3, and when applicable the control group (Model 2) were used as the baseline. Since there were four models on the same data set, the Benjamini–Hochberg correction was used to account for multiple testing [21]. Adjusted *p*-values < 0.05 were considered significant. All models are described in detail in the Supplementary Materials.

## 3. Results

### 3.1. Study Population

Between September 2019 and June 2021, 26 patients with TAAA Crawford Type I, II and III were screened for ER; 2 patients were excluded due to ‘shaggy aorta’ and malignancy. Of the 24 patients included in the PAPA ARTiS Trial, 1 died before ER due to non-aneurysm-related death, and 3 patients refused to receive HSI, rendering 20 patients in the HSI study (Figure 1). A total of 10 (50.0%) patients were randomized to the intervention group, while the other 10 (50.0%) patients were included in the control group. The mean age of the study population was 66.5 ± 10.2 years, with 45.0% of patients identifying as female. Cardiovascular risk factors were common, with hypertension present in 95.0% and hyperlipidemia in 60.0% of patients. Additionally, 80.0% of patients reported being current or former smokers. The demographics are summarized in Table 1.

### 3.2. Aneurysm Characteristics

Most patients (85.0%) were treated for TAAA Crawford type II, with a mean maximal aortic diameter of 59.7 ± 9.8 mm. Two patients (10.0%) had associated common iliac artery aneurysm on one side. Thirteen patients (65.0%) received previous repair of the aorta at a median of 37.3 months (range, 1–208 months). On thin-sliced computer tomography angiography (CTA), an average of 17.3 ± 5.1 patent SAs (range, 8–25) were identified in the area to be covered by stentgraft. All subclavian arteries were patent, 90.0% of right hypogastric arteries, and 95.0% of left hypogastric arteries.

### 3.3. Control Group

Nine patients in the control group received staged ER, with one patient treated in a single session. Six patients received two-stage ER, two patients were treated in three stages, and one patient in five stages due to associated common iliac aneurysm (Supplemental Table S1). Staging was performed with implantation of combinations of off-the-shelf thoracic stentgrafts, fenestrated/branch custom-made stentgrafts, and bifurcated stentgrafts. In total, 24 sessions of stentgraft implantation were performed with a mean time interval of 41.75 ± 11.62 days (range, 27–68) between procedures, all via percutaneous transfemoral access. HSI was performed at T1 and T3 for all patients (24 measurements per region). Two patients declined at T2 and T4, resulting in 22 measurements per region. At T5, 9 measurements per region were taken (Supplemental Table S2).

### 3.4. Intervention Group

In the intervention group, two patients received one session of MIS2ACE; five patients received two sessions; two patients received three sessions; and in one patient, four sessions of MIS2ACE were performed (Supplemental Table S3). The median time interval between sessions was 16 days (range, 5–40 days). A total of 70 SAs and 1 inferior mesenteric artery were coiled, averaging 3.18 ± 1.10 SAs (range, 1–5 SAs) per session. Of the open SAs in the MIS2ACE group, 41.42% were coiled with a technical success rate of 100%. All MIS2ACE sessions were performed in local anesthesia. HSI was performed at T1 and T2 for all 22 sessions, with 21 measurements at T3 and 13 at T4; 7 measurements were taken at T5, totaling 85 measurements per region for analysis (Supplemental Table S4). The mean interval between the last session of MIS2ACE and the first implantation stentgraft was 34.00 ± 18.89 days, and the mean time to complete aneurysm exclusion was 72.40 ± 29.8 days. In the intervention group, five patients received staged ER: three received two sessions, one patient received three sessions, and one patient received four sessions of stentgraft implantation. Thoracic stentgrafts, fenestrated/branch aortic stentgrafts, bifurcated stentgrafts and iliac branch devices were implanted. In total, 18 sessions were performed after MIS2ACE, all via percutaneous transfemoral access. The number of HSI measurements performed at each time is shown in Supplemental Table S4. The intervention characteristics are presented in Supplemental Table S5.

### 3.5. Model 1

**Figure 3:**
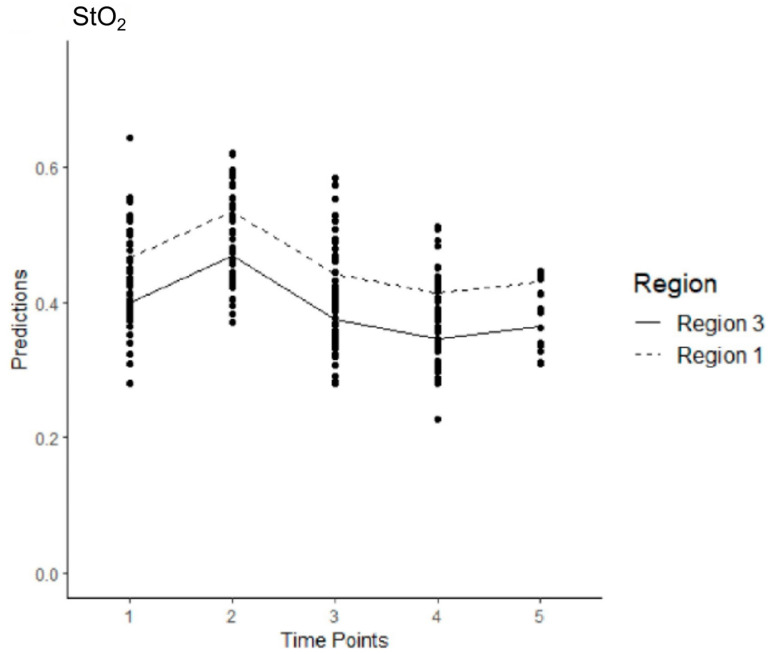
Estimated levels of StO_2_ across time and region after all the sessions of stentgraft implantation in the control group (Model 1). The mean values are connected with the lines.

In Model 1, 202 observations were collected across five time points for both regions (Supplemental Table S2). The average StO_2_ level in R3 at T1 was estimated at 0.399. The effect of changing from T1 to any of the other time points was significant, with a positive effect from changing from T1 to T2 (*p* < 0.001) and negative effects from T1 to T3 (*p* = 0.040), T4 (*p* < 0.001) or T5 (*p* = 0.028). The effect of changing from Region 3 to Region 1 was significant and positive, with StO_2_ levels expected to increase by 0.067 (*p* < 0.001) (Figure 3).

### 3.6. Model 2

**Figure 4:**
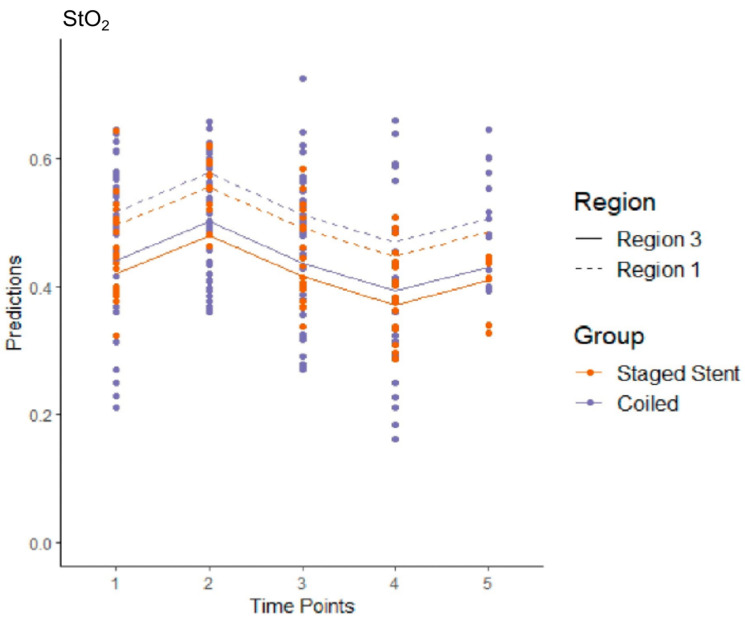
Estimated levels of StO_2_ across time and region after MIS2ACE in the intervention group and after the implantation of the first stentgraft in the control group (Model 2). The mean values are shown with connecting lines.

Ten patients were included from the intervention group and nine from the control group, with one patient being excluded due to receiving only one stent. This resulted in 170 measurements for the intervention group and 78 measurements for the control group over five time points for both regions. For StO_2_, there was no significant difference between the implantation of one or more coils and the implantation of the first stentgraft (*p* = 0.491). The effect of changing from R3 to R1 was significant (*p* < 0.001), and StO_2_ levels were expected to increase by 0.076 on average when changing from R3 to R1. The effect of changing from T1 to T2 (*p* < 0.001) or T4 (*p* < 0.001) was statistically significant. At T2, the StO_2_ levels were expected to increase by 0.061 compared to T1. At T4, the StO_2_ levels were expected to decrease by 0.048 on average compared to the reference T1. The effect of changing from T1 to T3 (*p*= 0.651) or T5 (*p* = 0.481) was not significant (Figure 4).

### 3.7. Model 3

**Figure 5:**
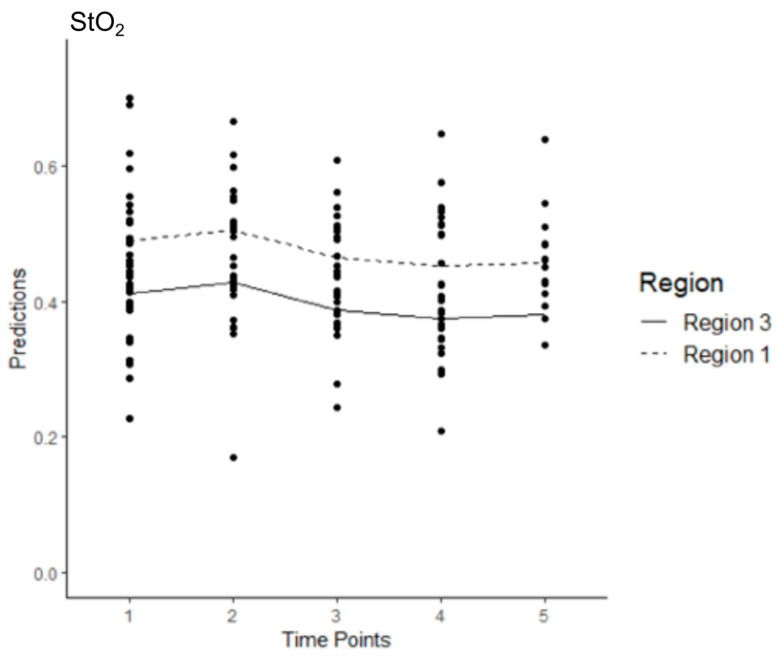
Estimated levels of StO_2_ across time and region after all the sessions of stentgraft implantation in the intervention group (Model 3). The mean values are shown with connecting lines.

In Model 3, 142 observations were collected across five time points for both regions. The average StO_2_ level in R3 at T1 was estimated at 0.412. The effect of changing from T1 to any of the other time points was not significant except for T4, with the effect of changing from T1 to T4 being negative. The effect of changing from R3 to R1 was significant and had a positive effect on the StO_2_ levels. The StO_2_ level was expected to decrease by 0.037 at T4 compared to the StO_2_ level at T1, T2 (*p* = 0.346), T3 (*p* = 0.111), T4 (*p* = 0.021) and T5 (*p* = 0.130). There was a significant positive effect for R1 compared to R3; the StO_2_ levels were expected to increase by 0.077 when changing from R3 to R1 (*p* < 0.001) (Figure 5).

### 3.8. Model 4

**Figure 6:**
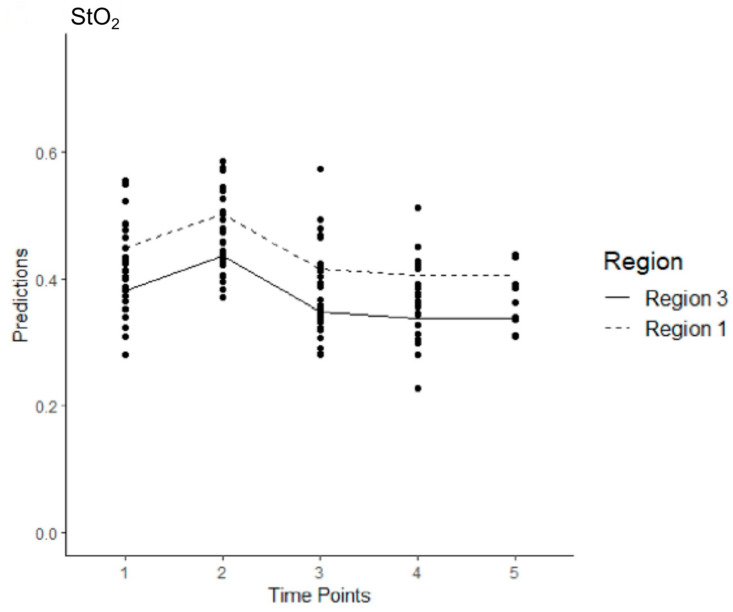
Estimated levels of StO_2_ across time and region after all the sessions of stentgraft implantation in the control group (Model 4). The mean values are shown with connecting lines.

In Model 4, 124 observations were collected across five time points for both regions. The average StO_2_ level in R3 at T1 was estimated at 0.382. The effect of changing from T1 to any of the other time points was significant, with the effect of changing from T1 to T2 being positive (*p* < 0.001) and all the effects of changing from T1 to T3 (*p* = 0.008), T4 (*p* < 0.001) or T5 (*p* = 0.019) being negative. There was a significant positive effect for R1 compared to R3; the StO_2_ levels were expected to increase by 0.067 when changing from R3 to R1 (*p* < 0.001) (Figure 6).

## 4. Discussion

Monitoring spinal cord perfusion during and after TAAA repair is crucial for early diagnosis of post-treatment SCI. Currently, there are limited methods for the detection of SCI outside the operating room. NIRS has demonstrated the ability to assess tissue oxygenation in the paraspinous vasculature in porcine models, showing that the oxygenation status of the regional paraspinous musculature correlates with spinal cord tissue oxygenation [13]. As a result, NIRS has been introduced as a method for monitoring spinal cord perfusion during and after AR. In humans undergoing open TAAA repair, continuous NIRS and MEP measurements suggested an association, and NIRS demonstrated a direct response to compromised aortic blood flow during aortic cross-clamping, further validating the use of NIRS for spinal cord perfusion monitoring [22,23]. However, despite the real-time and non-invasive nature of NIRS measurements, these are limited to the specific area of the optodes.

This limitation may be addressed by HSI, which offers data on StO_2_ from an adjustable measurement area. Additionally, HSI can capture a broader range of wavelengths compared to NIRS, making it less susceptible to errors caused by scattering or other tissue pigments by minimizing constant and linear influences on measurements [16].

In our pilot study, we measured StO_2_ above the paraspinous musculature with HSI before and up to 10 days after the staged ER of TAAA with MIS2ACE and stentgrafting. A total of 514 HSI measurements were analyzed using four models, each addressing a specific question.

Model 1 aimed to assess the feasibility of HSI to monitor StO_2_ after standard ER of TAAA. Stentgraft implantation significantly affected StO_2_ at all time points, showing an initial increase in StO_2_, followed by a decrease after 5 days and then a rise without returning to the baseline by day 10. These results suggest that HSI is sensitive to changes in StO_2_ and can, like NIRS, be used to monitor spinal cord perfusion during and after ER.

Model 2 aimed to measure the effect of priming the CN on StO_2_ values. No significant difference in StO_2_ was found between one or more coil implantations and the first stentgraft. However, both methods significantly affected StO_2_ during follow-up: StO_2_ increased immediately, decreased at 5 days and returned to baseline by 10 days in both groups. Therefore, priming of the CN appears to impact spinal cord perfusion, with the effect being reversible. These findings suggest that the CN is able to build new arterioles and recruit larger preexisting arteries when exposed to ischemia.

In Models 3 and 4, we evaluated the efficacy of MIS2ACE and stentgraft implantation as priming methods, examining their impact on StO_2_ changes during ER of TAAA. In the intervention group, a significant StO_2_ decrease at 5 days was followed by a return to baseline values by 10 days, suggesting that the CN compensated for the loss of direct blood flow. In contrast, in the control group a significant increase in StO_2_ immediately post-intervention was followed by a decrease after 24 h, with no recovery to baseline after 5 or 10 days. This indicates a prolonged perfusion deficit of the paraspinous musculature and the spinal cord. Thus, priming the CN with MIS2ACE allowed for StO_2_ recovery, unlike stentgraft priming, highlighting the differences in efficacy between these two priming methods.

This pilot study is the first to use HSI for monitoring paraspinous oxygenation up to 10 days after ER of TAAA, making a direct comparison with existing data challenging. The initial increase in StO_2_ observed immediately after each procedure may be explained by compensatory blood flow to the CN [7]. Previous anatomical studies in the porcine model have described steal phenomena, with bidirectional blood flow to and from the CN, which may explain this observation [24]. Additionally, baseline StO_2_ values were significantly higher in the thoracic compared to the lumbar region in all models. This is likely due to collateralization of the CN and the input from subclavian and internal thoracic arteries at the thoracic level, as described previously in animal models [13].

In a porcine model, the remodeling of the CN following SA occlusion was examined over a period of 5 days, revealing significant anatomical changes [8]. Within the first 24 hours, enlargement of the arteries was observed, with CN remodeling being completed after 5 days with dramatic shifts in the distribution of intramuscular vessel diameters in the paraspinous CN [8]. In a similar model with only intra-procedural monitoring, NIRS demonstrated a decrease in signal after 10 minutes, followed by a return to baseline within 20 minutes, most likely due to redistribution and not remodeling [15].

In contrast to these findings in animal models, our data suggest that total recovery of the CN takes longer than 5 days, with baseline StO_2_ values only being reached after 10 days in patients primed with MIS2ACE. These findings indicate that MIS2ACE had a protective effect, induced remodeling and effectively primed the CN. This could influence the interval between sessions of staged ER of TAAA, potentially reducing SCI risk and improving outcomes, with the current standard being 7 days [10]. In contrast, patients who did not undergo MIS2ACE still reported reduced StO_2_ values after 10 days, suggesting that stentgrafting alone might be insufficient for CN priming.

### Limitations

The main limitation of this proof-of-concept study is the small sample size and the lack of a control group without intervention to validate our findings. It is unclear whether changes in StO_2_ are due to ischemia or physiological remodeling, as no predefined cut-off values have been established. HSI limitations include interference from naevi, fat tissue, skin thickness and the inclusion of only light-skinned patients. As this was the first in-human study to observe the impact of staged TAAA repair up to 10 days post-procedure with HSI, further studies with larger sample sizes and longer follow-up periods are needed to explore CN remodeling following ER of TAAA.

## 5. Conclusions

HSI effectively detects changes in StO_2_ above the paraspinous musculature after ER of TAAA. Both methods of CN priming had no lasting effect on StO_2_, indicating their safety. StO_2_ significantly decreased after ER of TAAA over a period of 10 days in the control group but not in the intervention group, demonstrating the efficacy of MIS2ACE for priming the CN.

## Figures and Tables

**Table 1:** Patients’ demographics and aneurysm characteristics.

		Intervention Group (*n* = 10)	Control Group (*n* = 10)	Total (*n* = 20)
Variables		No. (%)	No. (%)	No. (%)
Sex				
Male		5 (50)	6 (60)	11 (55)
Female		5 (50)	4 (40)	9 (45)
Age (years)				
Mean ± SD		68.3 ± 8.4	64.6 ± 11.4	66.5 ± 10.2
Median (range)		70.5 (51–80)	65.5 (42–81)	67.0 (42–81)
History of hypertension		10 (100)	9 (90)	19 (95)
COPD		3 (30)	3 (30)	6 (30)
Smoker		7 (70)	9 (90)	16 (80)
Coronary heart disease		3 (30)	3 (30)	6 (30)
Diabetes mellitus		0 (0)	2 (20)	2 (10)
Renal insufficiency				
GFR < 60 mL/min/1.73 m^2^		3 (30)	5 (50)	8 (40)
Mean GFR ± SD		72.1 ± 18.6	58.7 ± 26.2	65.4 ± 23.2
Hyperlipidemia		7 (70)	5 (50)	12 (60)
Body mass index (BMI)				
Mean ± SD		27.3 ± 4.9	29.3 ± 6.8	28.5 ± 6.0
Median (range)		26.3 (21.5–40.6)	25.2 (23.5–41.9)	25.8 (21.5–41.9)
ASA Classification				
II		2 (20)	2 (20)	4 (20)
III		6 (60)	6 (60)	12 (60)
IV		2 (20)	2 (20)	4 (20)
Crawford Classification				
Type II		9 (90)	8 (80)	17 (85)
Type III		1 (10)	2 (20)	3 (15)
Maximal Aortic Diameter, mm				
Mean ± SD		58.5 ± 11.0	61.0 ± 8.8	59.7± 9.8
Previous Aortic Repair		5 (50)	8 (80)	13 (65)
Ascending Aortic Repair		3 (30)	5 (50)	8 (40)
	ET	1 (10)	3 (30)	4 (20)
	FET	2 (20)	4 (40)	6 (30)
TEVAR		1 (10)	1 (10)	2 (10)
EVAR		1 (10)	1 (10)	2 (10)
Etiology				
Degenerative		6 (60)	7 (70)	13 (65)
Post-dissection		4 (40)	3 (30)	7 (35)
Segmental Artery to be covered by the stentgraft				
Total		159	188	347
Mean ± SD		15.9 ± 5.3	18.8 ± 4.6	17.3 ± 5.1

Continuous data presented as mean ± standard deviation; categorical data given as counts. COPD: chronic obstructive pulmonary disease; GFR: glomerular filtration rate; ASA: American Society of Anesthesiologists; TEVAR: thoracic endovascular aortic repair; FET: frozen elephant trunk.

## Data Availability

The data presented in this study are available on request from the corresponding author due to privacy restrictions.

## Supplementary Materials

The following Supporting Information can be downloaded at: https://www.mdpi.com/article/10.3390/jcm14093188/s1, Supplemental Table S1. Staging the endovascular procedures during the endovascular repair of thoracoabdominal aortic aneurysm in the control group, Supplemental Table S2. Hyperspectral imaging measurements during the endovascular repair of thoracoabdominal aortic aneurysm in the control group, Supplemental Table S3. Staging the endovascular procedures during the endovascular repair of thoracoabdominal aortic aneurysm in the intervention (MIS2ACE) group, Supplemental Table S4. Hyperspectral imaging measurements during the endovascular repair of thoracoabdominal aortic aneurysm in the intervention (MIS²ACE) group, Supplemental Table S5. Characteristics of the interventions in both the control and the intervention group.

## Abbreviations

The following abbreviations are used in this manuscript:

AR	Aortic Repair
CN	Collateral Network
CTA	Computer Tomography Angiography
ER	Endovascular Repair
HSI	Hyperspectral Imaging
MIS2ACE	Minimally Invasive Segmental Artery Coil Embolization
NIRS	Near-Infrared Spectroscopy
NIR PI	Near-Infrared Perfusion Index
SA	Segmental Artery
SCI	Spinal Cord Ischemia
StO_2_	Tissue Oxygenation
TAAA	Thoracoabdominal Aortic Aneurysm
THI	Tissue Hemoglobin Index
TWI	Tissue Water Index

## References

[1] Stoecker J.B., Wang G.J. (2021). Epidemiology of thoracoabdominal aortic aneurysms. Semin. Vasc. Surg..

[2] Kim J.B., Kim K., Lindsay M.E., MacGillivray T., Isselbacher E.M., Cambria R.P., Sundt T.M. (2015). Risk of Rupture or Dissection in Descending Thoracic Aortic Aneurysm. Circulation.

[3] Ellahi A., Shaikh F.N., Kashif H., Khan H., Ali E., Nasim B., Adil M., Huda Z., Liaquat A., Arshad M.S. (2022). Effectiveness of endovascular repair versus open surgery for the treatment of thoracoabdominal aneurysm: A systematic review and meta analysis. Ann. Med. Surg..

[4] Miranda V., Sousa J., Mansilha A. (2018). Spinal cord injury in endovascular thoracoabdominal aortic aneurysm repair: Prevalence, risk factors and preventive strategies. Int. Angiol..

[5] Bisdas T., Panuccio G., Sugimoto M., Torsello G., Austermann M. (2015). Risk factors for spinal cord ischemia after endovascular repair of thoracoabdominal aortic aneurysms. J. Vasc. Surg..

[6] Rizza A., Trimarchi G., Di Sibio S., Bastiani L., Murzi M., Palmieri C., Foffa I., Berti S. (2023). Preliminary Outcomes of Zone 2 Thoracic Endovascular Aortic Repair Using Castor Single-Branched Stent Grafts: A Single-Center Experience. J. Clin. Med..

[7] Griepp E.B., Di Luozzo G., Schray D., Stefanovic A., Geisbüsch S., Griepp R.B. (2012). The anatomy of the spinal cord collateral circulation. Ann. Cardiothorac. Surg..

[8] Etz C.D., Kari F.A., Mueller C.S., Brenner R.M., Lin H.M., Griepp R.B. (2011). The collateral network concept: Remodeling of the arterial collateral network after experimental segmental artery sacrifice. J. Thorac. Cardiovasc. Surg..

[9] O’Callaghan A., Mastracci T.M., Eagleton M.J. (2015). Staged endovascular repair of thoracoabdominal aortic aneurysms limits incidence and severity of spinal cord ischemia. J. Vasc. Surg..

[10] Branzan D., Etz C.D., Moche M., Von Aspern K., Staab H., Fuchs J., Bergh F.T., Scheinert D., Schmidt A. (2018). Ischaemic preconditioning of the spinal cord to prevent spinal cord ischaemia during endovascular repair of thoracoabdominal aortic aneurysm: First clinical experience. EuroIntervention.

[11] Tenorio E.R., Ribeiro M.S., Banga P.V., Mendes B.C., Kärkkäinen J., DeMartino R.R., Oderich G.S. (2022). Prospective Assessment of a Protocol Using Neuromonitoring, Early Limb Reperfusion, and Selective Temporary Aneurysm Sac Perfusion to Prevent Spinal Cord Injury During Fenestrated-branched Endovascular Aortic Repair. Ann. Surg..

[12] Jacobs M.J., Mess W., Mochtar B., Nijenhuis R.J., van Eps R.G.S., Schurink G.W.H. (2006). The value of motor evoked potentials in reducing paraplegia during thoracoabdominal aneurysm repair. J. Vasc. Surg..

[13] von Aspern K., Haunschild J., Hoyer A., Luehr M., Bakhtiary F., Misfeld M., Mohr F.W., Etz C.D. (2016). Non-invasive spinal cord oxygenation monitoring: Validating collateral network near-infrared spectroscopy for thoracoabdominal aortic aneurysm repair. Eur. J. Cardiothorac. Surg..

[14] von Aspern K., Haunschild J., Ziemann M., Misfeld M., Mohr F.W., Borger M.A., Etz C.D. (2019). Evaluation of collateral network near- infrared spectroscopy during and after segmental artery occlusion in a chronic large animal model. J. Thorac. Cardiovasc. Surg..

[15] von Aspern K., Haunschild J., Heier M., Ossmann S., Mohr F.W., A Borger M., Etz C.D. (2021). Experimental near-infrared spectroscopy- guided minimally invasive segmental artery occlusion. Eur. J. Cardiothorac. Surg..

[16] Lu G., Fei B. (2014). Medical hyperspectral imaging: A review. J. Biomed. Opt..

[17] Petroff D., Czerny M., Kölbel T., Melissano G., Lonn L., Haunschild J., Etz C.D. (2019). Paraplegia prevention in aortic aneurysm repair by thoracoabdominal staging with ‘minimally invasive staged segmental artery coil embolisation’ (MIS2ACE): Trial protocol for a randomised controlled multicentre trial. BMJ Open.

[18] Holmer A., Marotz J., Wahl P., Dau M., Kämmerer P.W. (2018). Hyperspectral imaging in perfusion and wound diagnostics-methods and algorithms for the determination of tissue parameters. Biomed. Tech..

[19] R Core Team R. (2022). A Language and Environment for Statistical Computing.

[20] Bates D., Mächler M., Bolker B., Walker S. (2015). Fitting Linear Mixed-Effects Models Using lme4. J. Stat. Softw..

[21] Benjamini Y., Hochberg Y. (1995). Controlling the False Discovery Rate: A Practical and Powerful Approach to Multiple Testing. J. R. Stat. Soc. Ser. B Methodol..

[22] Boezeman R.P., van Dongen E.P., Morshuis W.J., Sonker U., Boezeman E.H., Waanders F.G., de Vries J.-P.P. (2015). Spinal Near-Infrared Spectroscopy Measurements During and After Thoracoabdominal Aortic Aneurysm Repair: A Pilot Study. Ann. Thorac. Surg..

[23] Etz C., von Aspern K., Gudehus S., Luehr M., Girrbach F., Ender J., Borger M., Mohr F. (2013). Near-infrared Spectroscopy Monitoring of the Collateral Network Prior to, During, and After Thoracoabdominal Aortic Repair: A Pilot Study. Eur. J. Vasc. Endovasc. Surg..

[24] Etz C.D., Kari F.A., Mueller C.S., Silovitz D., Brenner R.M., Lin H.M., Griepp R.B. (2011). The collateral network concept: A reassessment of the anatomy of spinal cord perfusion. J. Thorac. Cardiovasc. Surg..

