# Supplementary material for: Evaluation of Spinal Cord Blood Supply with Hyperspectral Imaging of the Paraspinous Musculature During Staged Endovascular Repair of Thoracoabdominal Aortic Aneurysm: A Sub-Study of the Prospective Multicenter PAPA-ARTiS Trial
Source: J Clin Med. 2025 May 5;14(9):3188. doi: 10.3390/jcm14093188 (PMC12072341; doi:10.3390/jcm14093188)
Supplement: Supplementary file 1 [file jcm-14-03188-s001.zip › jcm-3529391-supplementary.pdf]

Supplemental Material

Supplemental Table S1: Staging the endovascular procedures during the endovascular repair of thoracoabdominal aortic aneurysm in the control group

	**Single-stage Stentgrafting**	**Staged Stentgrafting**		
	Patients (n)	Patients (n)	Stentgraft Sessions/ Patient	Sessions of Stentgrafting (n)
	1	6	2	12
		2	3	6
		1	5	5
**Total**	**1**	**9**		**23**

Supplemental Table S2: Hyperspectral imaging measurements during the endovascular repair of thoracoabdominal aortic aneurysm in the control group. *Including the patient that received only one session of stentgrafting.

	**First Stentgraft Implantation n = 9**		**Staged Stentgraft Implantation n = 15***	
	Observations (n)	Observation/Region (n)	Observations (n)	Observation/Region (n)
**T1**	18	9	30	15
**T2**	16	8	28	14
**T3**	18	9	30	15
**T4**	18	9	26	13
**T5**	8	4	10	5
**Total**	**78**	**39**	**124**	**62**

Supplemental Table S3: Staging the endovascular procedures during the endovascular repair of thoraco-abdominal aortic aneurysm in the intervention (MIS^2ACE) group.

	**MIS^2ACE**			**Stentgraft Implantation**		
	Patients (n)	MIS^2ACE Sessions/ Patient	MIS^2ACE Sessions (n)	Patients (n)	Stentgraft Sessions/ Patient	Sessions of Stentgrafting (n)
	2	1	2	5	1	5
	5	2	10	3	2	6
	2	3	6	1	3	3
	1	4	4	1	4	4
**Total**	**10**		**22**	**10**		**18**

Supplemental Table S4: Hyperspectral imaging measurements during the endovascular repair of thoracoabdominal aortic aneurysm in the intervention (MIS²ACE) group

	**MIS²ACE n = 22**		**Stentgraft Implantation n = 18**	
	Observations (n)	Observation/Region (n)	Observations (n)	Observation/Region (n)
**T1**	44	22	36	18
**T2**	44	22	28	14
**T3**	42	21	32	16
**T4**	26	13	32	16
**T5**	14	7	14	7
**Total**	**170**	**85**	**142**	**71**

Supplemental Table S5: Characteristics of the interventions in both the control and the intervention group. Mean ± SD.

	**Control Group Stentgraft Implantation (n = 24)**	**Intervention Group MIS²ACE Session (n = 22)**	**Intervention Group Stentgraft Implantation (n = 18)**
**Variables**			
Radiation dose (Gycm2)	283.7 ± 242.8	22.91 ± 9.93	220.3 ± 126.9
Radiation time (min)	27 ± 21.3	124.04 ± 70.97	31 ± 21.6
Length of covered aorta (mm)	472.9 ± 38.15		440.6 ± 98.35
Coverage of left subclavian artery (LSA)	0 (0%)		1* (10%)
ICU stay (days)	1.5 ± 2.3	0	2.0±1.1
Hospital stay (days)	8.0 ± 3.5	5.1 ± 2.8	9.6 ± 5.3

*The LSA was covered by the stentgraft and revascularized with a carotid-subclavian bypass at the end of the ER

Statistical Analysis

Model 1, 3 and 4

In Model 1,3 and 4 we fit the same equation on different data sets. In Model 1, we tested the feasibility of HSI in detecting StO2 changes (range, 0-1) during ER of TAAA, measurements acquired after stentgraft implantation without prior MIS2ACE were included. In Model 3, we assess StO2 changes during ER of TAAA after priming the CN with MIS2ACE, HSI measurements of all the sessions of implantation of stentgrafts in the MIS2ACE group were used. In Model 4 To measure the changes of StO2 after priming the CN with stentgrafts in the control group, HSI measurements from the following sessions were used.

We model StO2, y_{i,r_k,t_l}, where i denotes the individual patients $i = 1, \dots, n$; r_k the two regions with $k = 1{,}3$; and t_l the five categorical time points $l = 1, \dots ,5$. The random effects are denoted by b_i and assumed to be normally distributed ($b_i \overset{iid}{\sim} N(0, \tau^2)$). They represent the individual-specific deviation from the intercept, β_0, the baseline of the model, which contains the categories r_3, t_1, to which all other estimates (β_{r_1}, β_{t_2}, …, β_{t_5}) are compared.

The equation of this first model is

$$y_{i,r_k,t_l} = \beta_0 + \beta_{r_1} x_{i,\, r_1, t_l} + \beta_{t_2} x_{i,\, r_k, t_2} + \beta_{t_3} x_{i, r_k, t_3} + \beta_{t_4} x_{i, r_k, t_4,} + \beta_{t_5} x_{i, r_k, t_5,} + b_i + \epsilon_{i, r_k, t_l},$$

where the $x_{i,r_k,t_l,}$ represent the covariates and ϵ_{i,r_k,t_l} represents the error term of the model. The stage group, r_3 and t_1 are the reference categories and part of the intercept β_0.

Model 2

Model 2 aimed to measure the effect of priming the CN on StO2 values, all HSI measurements performed during MIS2ACE (intervention group), and the implantation of the first stentgraft (control group) were included.

We model StO2, y_{i,c_j,r_k,t_l}, where i denotes the individual patients $i = 1, \dots, n$; c_j denotes the type of treatment with j one of MISACE or staged stent; r_k the two regions with $k = 1{,}3$; and t_l the five categorical time points $l = 1, \dots ,5$. The random effects are denoted by b_i and assumed to be normally distributed ($b_i \overset{iid}{\sim} N(0, \tau^2)$). They represent the individual-specific deviation from the intercept, β_0, the baseline of the model, which contains the categories c_{stage}, r_3, t_1, to which all other estimates (β_{misace}, β_{r_1}, β_{t_2}, …, β_{t_5}) are compared.

The equation of this first model is

$$y_{i,c_j,r_k,t_l} = \beta_0 + \beta_{\text{misace}} x_{i,c_{\text{misace}},r_k,t_l} + \beta_{r_1} x_{i,c_j,r_1,t_l} + \beta_{t_2} x_{i,c_j,r_k,t_2} + \beta_{t_3} x_{i,c_j,r_k,t_3} + \beta_{t_4} x_{i,c_j,r_k,t_4,} + \beta_{t_5} x_{i,c_j,r_k,t_5,} + b_i + \epsilon_{i,c_j,r_k,t_l},$$

where the $x_{i,c_j,r_k,t_l,}$ represent the covariates and ϵ_{i,c_j,r_k,t_l} represents the error term of the model. The stage group, r_3 and t_1 are the reference categories and part of the intercept β_0.
